# A scientific approach to the reform of a medical curriculum: A personal account of the Vienna experience

**DOI:** 10.1007/s10354-018-0634-2

**Published:** 2018-04-12

**Authors:** Richard Marz

**Affiliations:** Teaching Center, Medical University of Vienna, Spitalgasse 23, 1090 Vienna, Austria

**Keywords:** Medical curriculum, Change management, Curriculum reform, Decision making, Curriculum reform process, Medizinisches Curriculum, Change Management, Studienplanreform, Entscheidungsfindung, Studienplanreformprozess

## Abstract

Planning and implementing a new curriculum at the Medical Faculty of the University of Vienna was a bold attempt to use a scientific approach. A curriculum of sequentially presented and departmentally controlled subject areas using oral examinations was replaced with horizontally and vertically integrated organ/function modules. The reform also introduced integrated written year-end examinations, a required research thesis, stronger clinical orientation starting already in the 1st semester and more elective components. The starting point, preparatory work, the legal framework, as well as the formal planning process from January 1998 until October 2001 are described and explained.

## Zusammenfassung

Die Planung und Implementierung eines neuen Studienplans an der Medizinischen Fakultät der Universität Wien war ein mutiger Versuch, einen wissenschaftlichen Zugang zu verwenden. Ein fachbasierter Studienplan mit mündlichen Einzelprüfungen durch die jeweiligen Institute bzw. Kliniken wurde durch ein modular aufgebautes, horizontal und vertikal integriertes System von Organ- bzw. Funktionseinheiten ersetzt. Die Reform beinhaltete auch schriftliche integrierte Jahresabschlussprüfungen, eine verpflichtende Diplomarbeit sowie eine Stärkung der klinischen Ausrichtung, die bereits mit dem 1. Studiensemester einsetzte, und mehr elektive Komponenten. Die Ausgangsbedingungen, Vorarbeiten, das gesetzgeberische Umfeld sowie die Planungsschritte vom Januar 1998 bis zur Implementierung im Oktober 2001 werden beschrieben und erklärt.

Education is governed by tradition and intuition [6]. It is hard to argue against this empirical observation but planning and implementing a new curriculum at the Medical Faculty of the University of Vienna^1^ was an audacious attempt to use a scientific approach. The formal planning process started in January 1998 and was completed with a pilot implementation in October 2001 and full implementation in October 2002. Preparatory work had started much earlier and, happily, modifications to the curriculum have been made almost every year since 2002. This paper references the old curriculum as the point of departure and focuses on the planning and decision-making process of the Vienna curriculum reform project until 2001.

The 20th century medical curriculum in Vienna, as in most Western countries, had its roots in the recommendations of the Flexner report from 1910 [7]. While a huge improvement at its introduction, by the second part of the century, with the vast expansion of medical knowledge as well as the changing expectations of society as to the role of a physician, more and more suggestions for new approaches to the education of medical students emerged [8]. These proposals addressed both the content of the curriculum as well as didactic methods which, in a nutshell, shifted emphasis from teaching to learning [9, 10]. There were even examples of dramatic changes to curricula being implemented at a few institutions around the world.

But few such innovative approaches were looked at seriously in Austria. Curricular changes were largely limited to introducing new subjects of instruction together with the corresponding oral examinations, though clinical training was also expanded. The overall structure of the curriculum did not change during the 20th century. It was based on the sequential contribution of individual departments: three phases going from pre-clinical subjects via bridging subjects such as pathology and pharmacology to clinical subjects. The examination system left much to students’ self-motivation: they were permitted to stand for an examination when they felt they were ready, with no penalty for postponing the day selected. With 23 examinations to pass to complete the requirements for an MD, by the 1990s what on paper was a 6-year curriculum took the successful student on average more than 9 years to complete. In addition, there was a high dropout rate at all stages of the curriculum.

A series of studies performed by Karl Krajic and his colleagues at the medical faculties of Vienna and Graz highlighted the many problems students faced in achieving satisfactory learning outcomes [11–13]. The main complaint was that graduates felt totally unprepared to start an internship or residency, which was the next step of their training. University teachers were characterized as “frustrationstolerant”: willing to subject themselves in their teaching activities to continuous failure. Most teachers were well aware that many students derived little or no meaningful learning from their efforts, yet kept on expending many working hours as well as the financial resources of the university on the same activities year after year [11]. But neither the Ministry of Higher Education, which paid for these studies, nor the faculties took any action.

Curricular change was difficult not only because of lack of interest among faculty members, it was also impeded by the administrative structure. The “Studienordnung”, the curricular framework, was the responsibility of the Austrian legislature and applied to all federal medical faculties (Vienna, Graz and Innsbruck)^2^ alike. The law prescribed all subjects of instruction, though not their contents, and established administrative regulations for everything from examination procedures to vacation times. The faculties had almost no latitude to design the actual curriculum, the “Studienplan”. A change to the Studienordnung was in principle possible, but in practice required agreement not only of all three faculties but also of a host of interest groups, foremost among which was the Ärztekammer, the chamber of physicians, thus ensuring that little real change could take place. Not surprisingly, a fairly ambitious attempt at curriculum reform failed in 1991. As a result, the only changes under discussion in the early 1990s were proposals to make both urology and orthopaedics separate subjects because the arrangement through which the surgery department was responsible for the teaching and examination of this content was considered unsatisfactory.

Academic reform efforts in Vienna in the 1980s and early 1990s had focused on experimentation with various innovative didactic methods since curriculum reform seemed out of reach [14–16]. While these add-on attempts were on the whole successful for improving the educational experience for the more motivated students, it became painfully obvious that this approach was unacceptable for most students, since it did not lead to immediate rewards, i.e. better preparation for the required examinations. Other efforts experimented with a new didactic tool: course evaluations and other types of evaluation [17–21]. But again, this turned out to be potentially useful but hardly the road to immediate curricular improvement [22]^3^. In hindsight, it was fortunate that an overly ambitious evaluation project was not funded, since it proposed to design curricular changes based on the outcome of course evaluations [23, 24].

The Graz Conferences^4^, Austrian conferences on medical education, in contrast, had a lasting impact. The first one took place in October 1995. Experts from the Netherlands, USA and Germany lectured and conducted workshops on topics such as quality considerations in higher education and various aspects of evaluation, while curriculum reform was not yet a topic [25, 26].

The legal framework changed completely with the passage of the UniStG (University Study Law; [27, 28]) which took effect in 1997, replacing the AHStG (Higher Education Study Law) from 1966 [29]. Now individual faculties were put in charge of planning and implementing their own curricula, thus dropping the requirement for common curricula at all Austrian institutions. The law also made it easy to make small changes to the curriculum once a year. While this law applied to all Austrian universities and all fields of study, the three medical faculties were the most innovative and radical in using this newly acquired freedom.

In Vienna, we were ready to grasp the chance to try for real curriculum reform. The impetus came from the “Mittelbau”—all faculty members below the level of full professor. This rank is formally recognized and organized in Austria, for instance, it has its own representation in the university senate. Until 2004, it also had an Austria-wide deliberative and representative body, the BUKO Medizinkommission^5^.

By the time of the second Graz Conference in the spring of 1997, the UniStG had been passed and the conference was given an agenda: to enable participants, who came from all three Austrian medical faculties, to become agents of real curricular change! The Graz conferences were working events with usable outcomes: in the early years, a typical conference workshop was led by one of the invited experts and lasted about 6 h [31, 32]. Although the UniStG gave each faculty the power to design their own curriculum, the outcomes were quite similar in Vienna, Graz and Innsbruck. This was largely the result of the many hours of discussion at the Graz Conferences. These Graz conferences have continued as yearly events, though the focus and the leadership has changed: in April 2018, the 22nd conference will take place in Maribor, Slovenia, and focus on future trends in medical education^6^.

The UniStG assigned all decision-making authority with regard to the curriculum to the “Studienkommission (StuKo)”, the directly elected curriculum commission for each field of study, rather than the faculty council. The StuKo for human medicine consisted of 12 members with 4 representatives from each of the main stakeholders (full professors, Mittelbau and students), whereas the former faculty council had more than 100 members in which full professors had 50% of the votes. The small size of the StuKo made it possible to have in-depth group discussions before decisions were taken. All curriculum-reform decisions, with only one exception, were unanimous!

The StuKo had the necessary curricular authority, but without a small group of dedicated faculty members from the Mittelbau and the explicit backing of the Dean, Wolfgang Schütz, not much would have happened. A taskforce was established to support the work of the StuKo, the MCW (Medizin Curriculum Wien), directed by Martin Lischka [33]^7^. Funding for this group came from the Ministry of Higher Education^8^ and from the Dean.

Discussions in the StuKo and among faculty members during 1997 had established a catalogue of problems relating to the curriculum:

- Too much theory and too few clinical skills. Students did not acquire meaningful clinical competence by the time of graduation.
- The duration of study was too long: the average (for students who ultimately graduated) was more than 9 years. The situation appeared even worse when the details were analysed: The first (entirely theoretical) part of the curriculum was supposed to last 4 semesters but on average it took 9 to complete. For the second part, the durations were 3 on paper and 5 in reality, with the two pathology examinations the major stumbling block. But the third, truly clinical part, was scheduled for 5 semesters and took only 4; apparently by this time students had become experts in gaming the system.
- Missing subject matter. Some examples: family medicine, nutrition, gerontology, medical genetics, gene therapy, psychosomatics, anaesthesia and intensive care, medical ethics, medical statistics, and medical economics.
- There was no enforceable requirement to acquire any research competencies and thus the scientific base of medicine was not adequately conveyed.

It became quite apparent that deep structural change to the curriculum was necessary to deal with at least some of the problems. Simple changes on the margins would not suffice. Further facts emerged:

- Resistance to any curricular change was enormous, both among faculty members and students. Initially, a change model of sequential modifications in small steps was considered, but it became clear that such an approach would grind to a halt very soon. Thus, revolution rather than evolution was necessary.
- The current model of departmental control of curricular content and examination requirements lay at the root of many curricular problems. Thus, some kind of content integration together with a centralized examination system would be necessary.

The first milestone was the development of a profile of student competencies for our graduates, which was approved by the StuKo on June 19, 1998 [33]. Accepted scientific criteria [34] were used, among them that the faculty must own the curriculum in order to convince other stakeholders of its soundness. Thus, as many stakeholders as possible were involved in an extensive discussion process to produce a product acceptable to a wide range of interest groups [33]. This profile still acts as the foundation and is published as an appendix to the curriculum [35]^9^.

Further steps along the reform path were:

- In the fall of 1998, a delegation of eight members of the StuKo and the taskforce visited the medical faculties in Liverpool and the Amsterdam Medical Center. Both institutions had recently made the move from a departmental to an integrated curriculum. This gave us first-hand information on how both faculty and students handled such a drastic transition and provided mainly our students with reassurance that such a change was indeed possible and desirable.
- The curriculum model was passed on January 21, 1999. This included horizontal and vertical integration of content, new examination procedures which replaced departmental oral examinations with integrated written year-end examinations, introduction of a required research thesis, stronger clinical orientation starting already in the 1st semester and more elective components.
- A rough draft of the curriculum, which included the new block and line components (mostly organ/function modules lasting 3–6 weeks supplemented with semester-long courses), was passed on June 25, 1999, and a revised version on October 29, 1999. This was the blueprint for the curriculum, which specified the sequence and duration, and as a consequence the number of credits, of each component. The components were given novel and rather generic names, for instance “From Molecule to Cell”^10^ to ensure a planning process without preconceived notions.
- The StuKo selected the coordinators for all modules of the new curriculum after an application process in January 2000. These coordinators had the responsibility of choosing an interdisciplinary planning team of at least six members, including faculty members from non-clinical as well as clinical subjects, a student and a physician from outside the university, preferably a general practitioner. These planning teams were charged with selecting the content which would be covered in their module as well as the appropriate didactic methods.
- The curriculum was passed in February 2001. It was implemented in a year-by-year fashion in a trial run for 150 students starting October 1, 2001^11^, and implemented for all beginning students starting October 1, 2002.

All decisions were made after consulting the literature and extensive discussions among ourselves and with international experts. The faculty at large was kept informed through frequently published blue-coloured MCW-NewsLetter(s), a website^12^ and discussion meetings, the “MCW Foren”. The publication *Tomorrows Doctors* by the British General Medical Council (GMC) had the most impact on the internal discussions. This publication had served as the starting point for a British curriculum reform process a few years earlier [36]. A paper referred to internally as the Manchester list [37] served as a guideline for integration and content selection.

The success of the new curriculum is evident [38, 39]. By imposing a strict schedule on instruction and examinations, students take on average less than 13 semesters to complete their studies. The student dropout rate has been greatly reduced and is mostly limited to the first year of instruction. All students are confronted with an introduction to medical statistics and have a research experience. The clinical orientation has been strengthened, though there is still considerable room for improvement. The year-end written examinations are a clear improvement over the previous system, but much remains to be done in this area as well.

However, curriculum reform must be viewed as a process, not as a finished product^13^. Though some reforms have been introduced recently—notably the 6th year of the curriculum has totally changed—after 20 years, it is time for a thoroughgoing review of the current curriculum.

Resistance to curriculum reform in Vienna moved through four phases:

1. A big reform is not necessary, small changes will suffice.
  Many hours of discussion were necessary to show that small changes, which left the general framework of the curriculum intact, would solve some problems only to worsen others.
2. Real reform is indeed necessary but impossible in Vienna.
  Resistance was finally overcome by presenting an outline of the new curriculum and pointing to examples of other universities that had undergone a similar curriculum change.
3. Reform is necessary, but to make it really good, we need more time for preparation and planning.
  A very difficult argument to counter, since nobody ever feels ready. But the StuKo made the decision to implement the reform, knowing that many details still needed to be worked out. However, a loss of momentum was considered the bigger risk.
4. Curriculum reform Is inevitable.
  This point of no return was impossible to predict, but in hindsight, there was a moment when even the hard-core gave up their resistance.

The question how a successful reform was possible in the face of inertia and strong resistance remains somewhat of a mystery. This has even been investigated by a group from Maastricht yielding few clear answers [40]. They do point to the new legal framework, but their thesis does not explain why other Austrian faculties took so little advantage of this opportunity. My best guess is that we had a critical mass of engaged colleagues with an ambitious vision who took pride in their work and could build on a democratic tradition that facilitated the bottom-up aspect of the process. The funding allowed employment of two full-time academics and a half-time administrative assistant for 1 year. Their work, together with the funded curriculum-reform proposal, established a road map which the reform process followed. Funding also paid for invitations to international experts like Geoff Norman to visit Vienna as well as travel to conferences and visits to universities in Great Britain, the Netherlands and Switzerland. The failure of the old curriculum was undeniable and the opponents of the reform were unable to offer a plausible alternative. The reform process had astute political leadership provided by the Dean and successive chairmen of the StuKo, Willi Firbas and Rudi Mallinger. Last but not least, we were supported by an exceptional international network of experts and a fair amount of luck also contributed.

## Footnotes

1 In 2004, the Medical Faculty of the University of Vienna was transformed by an act of the Austrian parliament into a new institution: The Medical University of Vienna. By that time, however, the planning process for the new curriculum had been completed and implementation had entered its final phase, so the institutional change had little effect on the process described here. But this change replaced the independent “Studienkommission” with a “Curriculumkommission” subordinate to the Senate, which will make future reforms on the scale described here much more difficult.

2 Private medical universities were not permitted in Austria until the 21st century.

3 A British student lamented about her experience: I was constantly asked to evaluate but some of my professors could not teach a dog how to sit (Times Higher Education supplement, August 1996).

4 http://grazconference.at/, http://www.oeghd.at/.

5 Medizinkommission der Bundeskonferenz des wissenschaftlichen und künstlerischen Personals. In this taskforce, the author encountered for the first time in Austria a group of colleagues who were willing to seriously consider didactic issues [30]. Foremost among them was Jörg Stein (Graz), who for many years co-organized the Graz Conferences with me. The group was chaired by Kurt Grünewald (Innsbruck), who a few years later became a member of the Austrian parliament.

6 http://grazconference.at/.

7 In addition, the taskforce consisted of an expert in higher education (Gottfried Csanyi), a recent graduate of the faculty (Patrick Merl), an administrative assistant (Anna Uhlich), an IT expert (Paolo Petta), and a member of the StuKo who was rapidly becoming an expert in Medical Education (Richard Marz). The web-based Curriculum Information System (CIS), a few years later rebranded and expanded as m3e, was initially the responsibility of Lucia Ucsnik, supported by Paolo Petta, Christian Holzbaur, and Richard Marz.

8 The funding was awarded for a curriculum-reform project proposal written and submitted by Richard Marz and Gottfried Csanyi.

9 https://www.meduniwien.ac.at/web/studium-weiterbildung/das-diplomstudium-humanmedizin/studienziel-qualifikationsprofil/.

10 From the beginning, Adi Ellinger was the coordinator of this unit.

11 Students were selected by lottery from the more than 300 applicants who wanted to be part of the pilot project.

12 The navigational design made by Paolo Petta is still in use today: https://studyguide.meduniwien.ac.at/curriculum/n202-2017/?state=0-77927-4747/diplomstudium-humanmedizin. The reform project website www.univie.ac.at/mcw was unfortunately lost during the transition medical faculty to medical university but fragments can be viewed via the waybackMachine http://archive.org/web/.

13 I have frequently quipped, if our reform held for another 100 years, it must be viewed as a total failure.
